# Effectiveness of clozapine, oxcarbazepine and rivastigmine combination in a bipolar disorder patient with initial cerebral atrophy

**DOI:** 10.1002/ccr3.2462

**Published:** 2020-01-07

**Authors:** Paolo Morana, Federico Mucci, Stefano Baroni, Alessandra Della Vecchia, Armando Piccinni, Benedetto Morana, Donatella Marazziti

**Affiliations:** ^1^ Casa di cura Morana Marsala Italy; ^2^ Dipartimento di Medicina Clinica e Sperimentale Section of Psychiatry University of Pisa Italy; ^3^ Brain Research Foundation (BRF) Lucca Italy; ^4^ UNICamillus University of health medical sciences Roma Italy

**Keywords:** bipolar disorders, clozapine, depression, fronto‐temporal atrophy, mixed features, oxcarbazepine, rivastigmine

## Abstract

This paper reports the case of a 46‐year‐old woman suffering from bipolar disorder of type I with mixed features with initial fronto‐temporal atrophy. Although considered treatment‐resistant to conventional strategies, she successfully responded to a combination of rivastigmine, clozapine, and oxcarbazepine.

## Graphical abstract

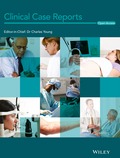

## 1 INTRODUCTION

Bipolar disorders (BDs) are psychiatric conditions characterized by mood, psychomotricity, and biorhythmicity disturbances. Bipolar disorder of type I (BDI) and of type II (BDII) represent the most severe types of BDs: the first is characterized by alternating depressive and manic episodes of great severity that frequently require hospitalization, while the second by depressive and hypomanic episodes.^1^ Currently, the debate concerning whether BDs should be primarily regarded as developmental or a neurodegenerative disorders is still open, although the two hypotheses do not appear necessarily mutually exclusive.^2^, ^3^ Indeed, *postmortem* studies highlighted different neuropathological abnormalities of glial and neuronal cells (in particular a loss of oligodendrocytes), albeit these alterations appear more limited than those observed in major neurodegenerative disorders.^3^ In any case, in the everyday clinical practice, the occurrence of neurodegenerative diseases (ie, Parkinson's disease, PD, Lewy body dementia, LBD, Alzheimer's disease, AD, fronto‐temporal dementia, FTD) in subjects with mood disorders is common, especially ‐ but not exclusively ‐ in elderly patients, and the implications of clinical and therapeutic management should not be considered negligible.^4^ To date, no disease‐modifying drug is available to stop or revert the neurodegenerative progression of the aforementioned diseases, so that the treatment is essentially empirical and based on symptomatic care.^4^ The presence of comorbidity with bipolar spectrum disorders (or other psychiatric conditions) further complicates the clinical picture, as well as possible drug interactions, so that the treatment of each individual case should be carefully tailored and personalized.^3^

Clozapine is the prototype of second‐generation antipsychotics (SGAs) commonly used in refractory psychoses and BD,^5^ while oxcarbazepine is prescribed as mood stabilizer in resistant BDI, although controlled studies are meager.^6^ The combination of clozapine and rivastigmine is considered as an effective symptomatic treatment in neurodegenerative disorders characterized by a possible underlying decreased functioning of the cholinergic system, such as PD, AD and LBD,^7^, ^8^ and even schizophrenia,^9^ but not considered a conventional intervention in BDI. The present paper reports the positive response of a patient suffering from BDI with mixed features and fronto‐temporal atrophy treated with an association of clozapine, oxcarbazepine, and rivastigmine.

## 2 CASE REPORT

Mrs. A. was a 46‐year‐old woman, housewife who had completed the high school, married with one daughter, with no personal history for substance or alcohol abuse, nor family history for any psychiatric disorders. She had been suffering from BDI since 20 years of age, when she presented the first severe depressive episode with mixed features, characterized by dysphoria and paranoid ideation requiring a hospitalization that led to a symptomatic improvement. However, after the discharge, she soon stopped the prescribed treatments and remained stable, until the subsequent mixed episode that occurred at the age of 25, after the birth of her daughter. Even in this case, although she was prescribed different treatments (consisting of mood stabilizers, such as lithium, valproic acid [VPA], carbamazepine, first‐generation antipsychotics [FGAs] and benzodiazepines [BDZs]), she had no compliance and, therefore, there was a recurrence of severe relapses every 2‐3 years, all requiring hospitalization. These episodes were mainly characterized by rapid alternations of depressed mood and dysphoria with agitation that were poorly responsive to drugs. In November 2018, she showed a severe depressive episode with mixed features (including dysphoria, auditory hallucinations, paranoid ideations and delusions of persecution, intermittent insomnia, and getaways from home) and was admitted to our psychiatry department. At the admission, she was diagnosed as BDI with mixed features, according to DSM‐5 criteria.^10^ The clinical assessment was carried out by means of Clinical Global Impression‐ Severity Scale (CGI‐S)^11^ and the Young Mania Rating Scale (YMRS)^12^: the patient's scores at the two scales were, respectively, 5 and 45. She showed alternating drowsiness and psychomotor agitation, associated with delusional ideation apparently not responsive to SGAs, such as quetiapine up to 200 mg/d, olanzapine up to 20 mg/d, and clozapine up to 200 mg/d that were sequentially prescribed for at least two weeks. Therefore, she was prescribed a combination of haloperidol (3 mg/d), paroxetine (20 mg/d), alprazolam (2 mg/d), VPA (1000 mg/d) and promazine (50‐150 mg/d *IM*). Since after five days there occurred a sudden onset of fever (38.5‐39°C), abnormalities of basal electroencephalography (EEG), specifically slow, often paroxysmal waves unresponsive to stimuli, and alterations of some blood chemistry tests (specifically, uricemia up to 8.10 mg/dL, C‐reactive protein [CRP] up to 15.6 mg/dL, white blood cells up to 14.000 and VPA levels up to 85.04 μg/dL), VPA and antipsychotics were stopped. However, several symptoms increased, in particular, psychomotor restlessness, delusions of persecution, auditory hallucinations, intermittent insomnia, confusion and loss of memory associated with paroxysmal episodes of tachycardia and blood pressure spikes.

In the next days, the fever decreased to 38°C, while CRP showed a further increase up to 19.9 mg/dL. Although no significant alterations were detected by the computerized tomography (CT) scan, a second EEG highlighted a worsening of the previous pattern. As the occurrence of degenerative encephalopathy was suspected by clinicians, all previous drugs were stopped and the patient was given BDZs (mainly alprazolam *per os* and diazepam *IV* when needed) and oxcarbazepine starting from 300 mg up to 1200 mg/d in one week. However, she showed no clinical improvement and a subsequent neurological assessment highlighted the presence of predominantly left mixed hypertonia and primitive reflexes (namely, frontal release signs and Myerson's sign), therefore, she was urgently transferred to the department of neurology.

After a magnetic resonance imaging (MRI) showing the presence of a “frontal‐temporal cerebral atrophy,” the patient underwent lumbar puncture with cerebrospinal fluid (CSF) proteomics research (ie, β‐amyloid, τ‐protein, and τ‐phosphorylated), and polymerase chain reaction (PCR) neurotropic viruses research (namely EBV, CMV, HSV‐1, HS EBV, CMV, HSV‐1, HSV‐2, HSV‐8‐2, HS‐8). All tests were normal, with the exception of β‐amyloid at the lower limits of the standard values and the detection of a mirror pattern (namely, the so‐called “focusing pattern IV”). Moreover, the patient performed a fluo‐deoxy‐glucose positron‐emission tomography (FDG‐PET) test that resulted normal, and an electrocardiography that showed only a minor increase of the QTc trait. Given the meager collaboration of the patient, the execution of neurocognitive tests was unsuccessful despite several attempts.

In the next two weeks, in spite of a reduction of inflammatory indexes, the overall clinical picture remained unchanged. Therefore, clozapine (150 mg/d,) was added in association with oxcarbazepine (1200 mg/d) and rivastigmine (4.25 mg/d, *transdermal*). An improvement of the sleep‐wake cycle, as well as of the drug compliance, was rapidly observed after three days, while confusion, delusional ideation, and psychomotor agitation underwent a slower reduction within the following two‐three weeks and disappeared in the next two months. No significant side effect was recorded.

The overall clinical picture resulted improved at the follow‐up after 10 months (CGI‐I score: 1 = much improved; YMRS score: 14), and particularly the mood resulted stabilized for the first time after almost 20 years, with a relative stability also of MRI abnormalities. Currently, the patient is taking the combinations of drugs at the same dosages prescribed when she was discharged from the hospital.

## 3 DISCUSSION

This case report highlights the challenge of BDI patients who do not respond to standard treatments, according to current international guidelines.^13^, ^14^, ^15^ Indeed, the pharmacological treatment of BDI is complex while encompassing a combination of mood stabilizers, such as lithium or anticonvulsants (VPA, carbamazepine, and lamotrigine), antipsychotics, antidepressants (ADs), and anxiolytics.^13^, ^14^, ^15^, ^16^ Both FGAs and SGAs are commonly used for manic episodes with a similar efficacy,^16^, ^17^ however, given the high risk of extrapyramidal side effects and depressive switches with FGAs, currently, these drugs are used as add‐on treatment in agitated patients requiring a rapid sedation, or alone in case of resistance to other previous treatments.^13^, ^14^, ^15^

The BDI patient described herein showed a good response to standard treatments after the hospitalization for the first depressed episode. However, she soon stopped all drugs and began to suffer every year from two to three episodes characterized by mixed features that were quite resistant to common therapeutic strategies, as it is generally the case.^17^, ^18^ Since during the last hospitalization she showed an elevated fever paralleled by alterations of unspecific peripheral inflammatory markers, as well as by EEG abnormalities suggestive of VPA intoxication, she underwent a MRI that revealed the presence of an initial frontal‐temporal cerebral atrophy, as supported also by lower CSF β‐amyloid concentrations.

Taken together, these findings suggested the presence of unspecific neuroinflammation processes that may constitute the common underpinnings of both severe mood symptoms and neurological abnormalities.^19^, ^20^ For these considerations and the difficult therapeutic management of the clinical picture, we treated the patient with a combination of clozapine, oxcarbazepine, and rivastigmine that provoked a progressive improvement along with the normalization of the inflammation indices and EEG abnormalities. Clozapine is widely used in resistant BDI,^5^, ^21^ but not included in the NICE guidelines, although recently some authors claimed that it should.^22^ Even oxcarbazepine is not yet included in the international guidelines for the treatment of BD, given the paucity of controlled studies,^6^ but it is used empirically in resistant cases or in patients who do not tolerate other mood stabilizers or carbamazepine that may provoke severe side effects and drug interactions.^23^ In our patient, oxcarbazepine was chosen for this reason, but also given the lack of previous response to other mood stabilizers and the sedating effect of this drug. Rivastigmine is a cholinesterase inhibitor widely employed in the treatment of mild to moderate AD and PD,^7^, ^8^, ^24^, ^25^ with a good efficacy in psychiatric symptoms frequently co‐occurring in these neurological disorders.^26^ In addition, scattered data would also suggest its possible use in visual hallucinations of schizophrenic patients.^9^, ^27^

The association of clozapine plus rivastigmine has been described as effective and safe in the scientific literature in neurodegenerative disorders, such as AD, PD, and LBD, where a possible deficit of the cholinergic system has been demonstrated or hypothesized.^7^, ^8^ In addition, cholinesterase inhibitors, such as rivastigmine, seem effective in resolving visual hallucinations, improving cognitive processes and controlling behavioral disturbances,^28^ as it happened in our patient.

To summarize, our case report would indicate that the combination of clozapine, oxcarbazepine, and rivastigmine appears effective and useful in resistant BDI, especially when accompanied by initial signs of brain atrophy.

The complexity of psychiatric and neurodegenerative disorders, together with the current limited knowledge of possible shared neuroinflammation underpinnings in these conditions, require further studies in this field that might lead to novel therapeutic strategies.

## CONFLICT OF INTEREST

Nothing to declare.

## AUTHOR CONTRIBUTIONS

Drs. Morana selected the patient. Drs. Marazziti, Mucci, Piccinni, Della Vecchia, and Baroni evaluated and assessed the clinical status of the patient during the different phases of the disorder. Dr P. Morana carried out the neurological assessment of the patient and prescribed all neurological tests. All the authors reviewed and selected the specific literature. Drs. Marazziti, Mucci, and P. Morana wrote the paper. All the authors reviewed the paper and agreed with the final version.
